# Worldwide circulation of HSV-2 × HSV-1 recombinant strains

**DOI:** 10.1038/srep44084

**Published:** 2017-03-13

**Authors:** David M. Koelle, Peter Norberg, Matthew P. Fitzgibbon, Ronnie M. Russell, Alex L. Greninger, Meei-Li Huang, Larry Stensland, Lichen Jing, Amalia S. Magaret, Kurt Diem, Stacy Selke, Hong Xie, Connie Celum, Jairam R. Lingappa, Keith R. Jerome, Anna Wald, Christine Johnston

**Affiliations:** 1 Department of Medicine, University of Washington, Seattle, WA 98195, USA; 2 Department of Laboratory Medicine, University of Washington, Seattle, WA 98195, USA; 3 Fred Hutchinson Cancer Research Center, Seattle, WA 98109, USA; 4 Department of Global Health, University of Washington, Seattle, WA 98195, USA; 5 Benaroya Research Institute, Seattle, WA 98102, USA; 6 Department of Infectious Diseases, University of Gothenburg, Guldhedsgatan 10B, 41346, Gothenburg, Sweden; 7 Department of Biostatistics, University of Washington, Seattle, WA 98195, USA; 8 Department of Epidemiology, University of Washington, Seattle, WA 98195, USA; 9 Department of Pediatrics, University of Washington, Seattle, WA 98195, USA.

## Abstract

*Homo sapiens* harbor two distinct, medically significant species of *simplexviruses*, herpes simplex virus (HSV)-1 and HSV-2, with estimated divergence 6–8 million years ago (MYA). Unexpectedly, we found that circulating HSV-2 strains can contain HSV-1 DNA segments in three distinct genes. Using over 150 genital swabs from North and South America and Africa, we detected recombinants worldwide. Common, widely distributed gene *UL39* genotypes are parsimoniously explained by an initial >457 basepair (bp) HSV-1 × HSV-2 crossover followed by back-recombination to HSV-2. Blocks of >244 and >539 bp of HSV-1 DNA within genes *UL29* and *UL30,* respectively, have reached near fixation, with a minority of strains retaining sequences we posit as ancestral HSV-2. Our data add to previous *in vitro* and animal work, implying that *in vivo* cellular co-infection with HSV-1 and HSV-2 yields viable interspecies recombinants in the natural human host.

Herpes simplex viruses types 1 and 2 are *Simplexviruses* in the *Alphaherpesvirinae* subfamily and are important pathogens with a natural host range restricted to humans. Herpesviruses have single-segment, linear DS DNA genomes and low mutation rates. Uniquely among primates, humans are known to harbor two *Simplexviruses,* each bearing homologous sets of approximately 74 open reading frames (ORFs). HSV-1/HSV-2 amino acid identity ranges from 43.5% for *US5,* encoding a glycoprotein, to 92% of *UL29*, encoding a DNA binding protein. It has been estimated that HSV-1 and HSV-2 diverged 6–8 million years ago^1^. HSV-1 shows orolabial tropism while HSV-2 causes most recurrent genital disease, although either can infect trigeminal or lumbosacral ganglia. Loci responsible for type-specific phenotypes can be mapped with HSV-1 × HSV-2 chimeras^2^,^3^. HSV-1 × HSV-2 recombination allows outgrowth of stable interspecies recombinant viruses (IRV) *in vitro*. Purposeful co-infection of animals with HSV-1 and HSV-2 can also yield recombinants, but they have not previously been reported in humans. Sequence patterns are consistent with recombination between HSV-1 strains, and between HSV-2 strains^4^, but there is little data concerning HSV-1 × HSV-2 IRV in the natural host. ChHV, isolated from *Pan trogdolytes*, is closer to HSV-2 than is HSV-1 across the genome, and is estimated to have diverged from HSV-2 < 2 million years ago^1^. It has been proposed that HSV-2 and ChHV have recombined^5^, but the contribution of intra- and inter-species recombination to contemporary HSV diversity is unclear.

Viral recombination can be clinically significant. Equine herpesvirus (EHV)-1 recombination with EHV-4 and EHV-9, distinct species, is associated with increased host range^6^. Vaccine × wild-type IRV occur in herpesviruses, parvoviruses, and coronaviruses^7^,^8^ and contribute to paralytic polio. Circulating, virulent strains of infectious laryngotracheitis virus, an alphaherpesvirus, descend from vaccine strains via recombination^8^,^9^. Replication-competent HSV-1 expressing GM-CSF is a licensed melanoma therapeutic, and HSV strains are in active study as vaccines, oncolytic, and gene therapy vectors^10^,^11^. The high worldwide seroprevalence of HSV-1 and HSV-2^12^ raises the possibility of recombination between these viruses and wild-type HSV, either within or across the HSV-1/HSV-2 divide.

We studied HSV-2 strains from North and South America and Africa, sequencing directly from genital swabs. We find that HSV-2 strains bear evidence of recombination events between HSV-1 and HSV-2 within genes *UL39, UL29,* and *UL30*. Most HSV-2 *UL29* and *UL30* sequences contain tracts of HSV-1 DNA, while rare specimens lack these HSV-1 recombination events. The HSV genes with interspecies recombination influence nucleotide metabolism, virulence, and sensitivity to antiviral drugs.

## Results

### Participants and specimens

We studied genital specimens from Seattle and Portland, USA, and sites in Peru and western, eastern, and southern Africa. The source natural history and clinical trials are detailed Methods. Initially, we newly analyzed swab specimens from unique persons by NGS (ref. 13, Genbank KX574860 through KX574908). After observing evidence of HSV-1 × HSV-2 IRV and confirmation by manual sequencing (below), we newly obtained *UL39* genotyping data from 111 other persons (Table 1). The criteria for additional specimen selection was to increase coverage to about 50 persons each in North and South American and Africa, and to maintain sex and HIV co-infection status distribution within geographic areas. Persons with HIV co-infection were relatively underrepresented in the US compared to South American and Africa. We used digital droplet PCR (ddPCR) (Supporting Information Methods) to genotype additional specimens. ddPCR was validated with specimens pre-sequenced by NGS and/or Sanger methods. The absolute fluorescence intensities for HSV-1 and HSV-2 genotypes varied between runs, but inclusion of samples with known genotypes in each ddPCR run permitted genotype assignment (examples, SI Fig. 1). One or both *UL39* ddPCR assays failed for 9% of genital swab DNA specimens, correlating with low amounts of HSV-2 DNA in a quantitative PCR test. Overall, we obtained unambiguous *UL39* genotyping data from 152 (91%) of 167 persons on whom it was attempted.

Our final data set (Table 1) incorporated all previously available *UL39* sequences (SI Table 1) with the NGS and ddPCR samples newly analyzed for this report. These sequences included sequence from 31 separate persons studied^14^ by NGS from cultured HSV-2 isolates, all with geographic information, but some lacking sex and/or HIV infection data. We added analysis of 3 laboratory strains, 2 strains from South Africa^15^, and 5 reported by Kolb *et al*.^16^ with limited demographics. Overall, the *UL39* analyses combined newly and previously reported from specimens from 193 persons, amongst whom 28%, 26%, and 37%, respectively, are from North America, South America, and Africa, with a few from Asia or of unknown origin. Men comprised 38% of the cohort with 50% women and 12% unknown, while 44% were HIV-1 infected, 45% HIV-1 uninfected, and 11% unknown.

### Detection of HSV-1 × HSV-2 IRV

*UL39* encodes the HSV large subunit of ribonucleotide reductase. We aligned 85 full-length HSV-2 *UL39* sequences, including present and prior NGS data^14^,^15^,^16^, Genbank, and in-house resequencing. The maximum nucleotide distance was 0.0183. Length varied from 1,141 to 1,146 AA/3,423 to 3,438 nucleotides (SI Table 2). Relative to the commonest 1,444 AA length in strain 186, strains SD90e^15^ and G had identical 3 AA deletions, strain HG52 had a 2 AA deletion, and Ugandan strain D39650^14^ had a 2 AA insertion. Indels were in the *UL39* N-terminus. HSV-1 *UL39* sequences (n = 82, Genbank) were each 1,137 AA long and had lower 0.006 aximum nucleotide distance. HSV-1 17+^17^ was used for further analyses.

**Figure 1:**
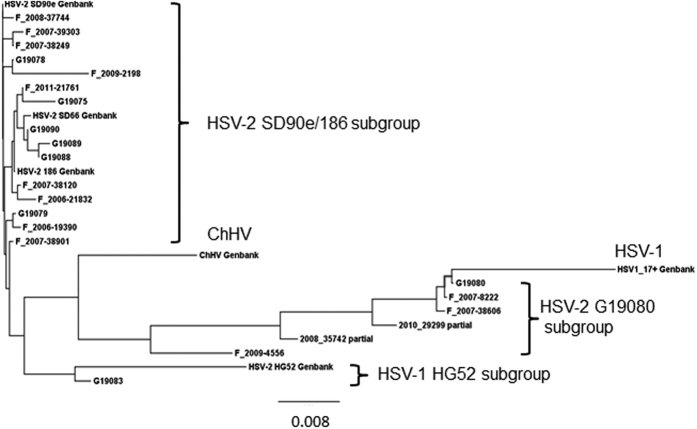
Phylogenetic tree of HSV-2 *UL39* sequences from bp 950 to the stop codon. The tree contains only one representative sequence for each unique nucleotide sequences in this region. Strain 333 is not included because it’s sequence is identical to SD90e in this region. Major subgroups, HSV-1 strain 17+, ChHV, and prototype strains from Genbank are identified. The largest HSV-2 group is contains the proposed prototype SD90e strain and strain 186, used for crossover analyses as described in the text.

We constructed *UL39* phylogenetic trees from HSV-2, HSV-1 17+ , and the ChHV homolog. Across the entire gene, the larger of the two major branches (SI Fig. 2) contained HSV-2 SD90e and laboratory strain 186. These data support the recent suggestion^15^,^18^ to use SD90e as the prototype HSV-2 strain, rather than strain HG52 (see below), and we named the largest *UL39* group SD90e/186. We did use strain 186 rather than SD90e for recombination analyses herein, because strain SD90e has a very rare 9 nucleotide deletion in *UL39* (above), while overall 81/85 strains (95%), including strain 186 had the same, prevalent length for *UL39*. Laboratory strain HG52 clearly fell in the minor *UL39* branch. HSV-1 and ChHV segregated outside of these major HSV-2 groups, with ChHV closer to HSV-2 than HSV-1 from HSV-2. Aside from low prevalence SNPs and short indels, we noticed that the prevalent differences between *UL39* sequences were concentrated in the 3′ region of the ORF. We therefore re-analyzed from base pair (bp) 950 to the stop codon (Fig. 1) to focus on the divergent region. To simplify, we present data for the 26 HSV-2 unique sequences in this region, excluding identical sequences. The HSV-2 SD90e/186 group was again the largest. The minor group now resolved more clearly into two subgroups. One subgroup, designated HG52, included 8 of 85 strains (9.4% of total). The second subgroup contained 15 of 85 (17.6%) sequences and was designated G19080 (SI Table 1) after a representative member. G19080 was isolated from a 45 year old HIV-1 co-infected US woman (Genbank KR135308^14^). Notably, both the HG52 and G19080 HSV-2 subgroups segregated with HSV-1, while ChHV again segregated separately.

**Figure 2:**
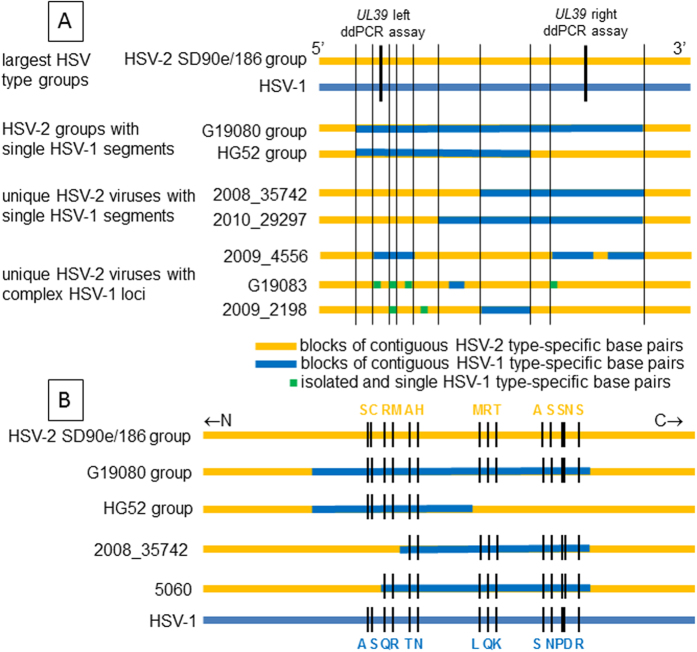
HSV *UL39* variants in circulating strains. Horizontal lines represent C-terminal *UL39* sequences to approximate scale. Group and virus names at left. (**A**) Genotypes. At top, the largest HSV-2 clade similar to strains SD90e and 186 (top) is yellow and the HSV-1 group is blue. Within IRV, blue bars represent zones of 4 or more contiguous HSV-1 variant SNPs. Green spots are isolated SNPs containing one HSV-1 variant nucleotide. Thin vertical black lines represent short crossover zones indistinguishable between HSV genotypes. Thick vertical black lines show approximate locations of type-specific ddPCR genotyping assays. (**B**) Amino acid variations in selected groups and viruses. Strains SD90e and 186 (top) are yellow and HSV-1 group (bottom) is blue. Blue bars in IRV represent zones of contiguous SNPS with HSV-1 variant nucleotides. Short black lines represent locations of color coded amino acid differences between strains.

Segregation of some HSV-2 *UL39* genes with HSV-1 (Fig. 1) could reflect continuous or discontinuous HSV-1 *UL39* alleles. Sequence alignments revealed blocks of contiguous HSV-1 markers in the HG52 and G19080 subgroups (Fig. 2A, SI Table 3). The G19080 subgroup contained a HSV-1 DNA segment of at least 457 bp as defined by flanking 5′ and 3′ HSV-1 alleles (Table 2). The flanking HSV-2 type-specific alleles were located 12 and 72 bp further in the 5′ and 3′ directions, respectively. The exact crossover loci were ambiguous given the zones of HSV-1/HSV-2 sequence identity between type-specific alleles.

Every HG52 and G19080 subgroup virus had identical 5′ and 3′ crossover boundaries. Remarkably, the 5′ crossover marks for the HG52 and G19080 IRV subgroups were also identical (Table 2). The HG52 subgroup included bp 2,572 of HSV-1 as the 3′ flanking HSV-1 allele, for a minimum 224 bp HSV-1 insert. These IRV had considerable *UL39* amino acid variation from strain 186 (Fig. 2B) in addition to nucleotide differences.

**Figure 3:**
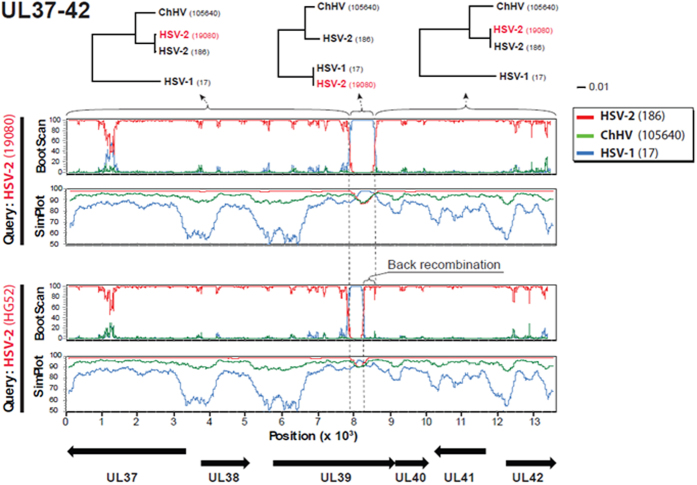
Recombination analysis of the *UL37* to *UL42* genomic region in HSV-2 strains 19080 (upper) and HG52 (lower). Bootscan and a Simplot analyses are depicted for each strain. At bottom the coding directions of the ORFs and length of the genomic region in kilobases from the stop codons in *UL37* and *UL42* are indicated. Clear shifts in bootstrap values supporting different phylogenetic topologies indicate recombination crossovers in the *UL39* gene in both strains (also indicated by vertical dotted lines). These crossovers are supported by the Simplot analysis, which demonstrates a shift in similarity with a higher similarity to HSV-1 in the recombination fragment. To further test and visualize ancestry of the recombination fragment in strain 19080, phylogenetic trees based on the recombination fragment and flanking regions are shown. Strain 19080 clearly clusters closely to HSV-1 in the tree based on the recombination fragment, and closely to HSV-2 in the flanking regions, further supporting recombination with HSV-2 as major parental, and HSV-1 as minor parental strains. The shorter recombination fragment in HSV-2 strain HG52 suggests a back recombination, where a strain with the larger HSV-1 recombination fragment has recombined again with another HSV-2 strain and expelled a part of the recombinant fragment.

We performed formal recombination analyses on HSV genome segments from *UL37* through *UL42* using HSV-1 17+ and HSV-2 186, G19080, and HG52 as representative strains. The bootscan algorithm readily identified crossover points with highly significant shifting (Fig. 3) coinciding with sequence inspection (SI Table 3). The three phylogenetic trees based on the recombinant fragment and the two flanking regions clearly demonstrated a shift in topology, where the recombinant HSV-2 strain 19080 clustered closely to HSV-1 in the tree representing the recombinant fragment, while clustering closely to the HSV-2 strain 186 in the flanking regions (Fig. 3). The high similarity between the HSV-2 strain 19080 and HSV-1 in the recombinant fragment was also shown by Simplot analysis. Similar results for a shorter recombinant fragment were observed for HSV-2 strain HG52 (Fig. 3). The phi-statistics for the presence of recombination had p-values of 0.0 for both HSV-2 strains G19080 and HG52, and the algorithms implemented in RDP4^19^ (i.e. RDP, GENECONV, bootScan, MaxChi, Chimaera, and SiScan) all yielded p-values supporting recombination (ranging from 2.7 × 10^−71^ to 3.2 × 10^−13^, and from 2.0 × 10^−41^ to 2.0 × 10^−07^ for G19080 and HG52, respectively). ChHV was included and no areas of significant recombination included this strain (Fig. 3).

### Rare *UL39* IRV genotypes

We used a ddPCR assay (loci schematized in Fig. 2) to rapidly genotype additional samples (Supplementary Methods, SI Table 4 for methods; sample data in SI Fig. 1). Strains HSV-1 17+ , HSV-2 G19080, HSV-2 HG52, and HSV-2 186 had the predicted HSV1:HSV1, HSV1:HSV1, HSV2:HSV1, and HSV2:HSV2 genotypes at the 5′ and 3′ *UL39* loci respectively. Most swab-derived DNA specimens had one of these genotype patterns. A new HSV1:HSV2 ddPCR pattern, consistent with additional *UL39* IRV, was detected in in two HIV-uninfected participants (samples 2010_29297 and 2008_35742) (Fig. 2, SI Fig. 1). For participant 5060 who donated sample 2010_29297, 11 swabs collected over 37 days had this ddPCR genotype.

Sanger sequencing (SI tables 5 and 6) confirmed the ddPCR data and defined two additional *UL39* IRV genotypes. Each had 3′ crossover markers identical to G19080 (SI Table 3, Fig. 2A) and novel 5′ crossovers internal to the long G19080 HSV-1 insert. Sample 2008_35742 had a longer HSV-1 insert than sample 2010_29297, containing 11 additional HSV-1 type-specific allelic variant markers and extending a minimum of 77 bp further in the 5′ direction within *UL39*. Bootscan, Simplot and phylogenetic analyses again were highly consistent with recombination (SI Fig. 3). Likely due to the relatively short region analyzed, the SiScan method failed to detect recombination in specimen 2010_29297 (participant 5060), although all other methods in RDP4 (p < 8.0 × 10^−5^) and the phi-statistics (p = 4.1 × 10^−7^) presented high statistical significance. Each RDP4 method (p < 1.5 × 10^−6^) and the phi statistics (p = 4.3 × 10^−9^) were significant for recombination for 2008_35742 (participant 6376). Taken together, the most parsimonious explanation for both the common HG52 group and these rare, short HSV-1 *UL39* fragments is an ancestral long recombination event giving rise to G19080, followed by back-recombination events wherein a part of the HSV-1 fragment in G19080 was expelled by recombination with a 186-like HSV-2 strain.

We noted additional unique sequences in the C-terminal *UL39* phylogeny (Fig. 1). Sequencing revealed three additional probable additional recombinants, each with HSV-1-like sequences remaining internal to the long G19080 HSV-1 insert (Supplementary Table 3), but with complex patterns. Sample 2009_2198 from an HIV-infected Zambian man and sample G19083 (ref. 14, from an HIV-uninfected US man) each had distinct sequence with four contiguous HSV-1 markers (blue bars in Fig. 2A). The 3′ crossover markers of the HSV-1 insert in 2009_2198 match the HG52 subgroup, suggesting common ancestry and a backcross. Sample 2009_4556 from an HIV co-infected Kenyan man has three regions of contiguous HSV-1 allelic markers. The 5′ end of 5′-most HSV-1 insert and the 3′ end of the 3′-most HSV-1 insert were identical to the HSV-1 flanking markers of G19080, suggesting common ancestry and backcrosses. Overall, we detected 6 distinct backcross patterns of HSV-1 × HSV-2 *UL39* recombination within the longest G19080 recombination event (Fig. 2A).

### Distribution of *UL39* variants

Evidence for geographically restricted HSV-2 genotypes is limited^14^,^20^. From 193 persons in this report and published data (Table 1)^14^,^15^,^16^, we excluded 8 published strains with uncertain geographic origin and one HIV co-infected and one HIV non-infected participant with evidence for dual strain infection (below), leaving 183 participants (Table 3). Excluding the 5 rare *UL39* genotypes noted above, 178 had mono-infection with a prevalent *UL39* variant (51 from North America, 49 from South America, 70 from Africa, and 8 from Asia). Each major *UL39* genotype was detected in Africa, North America, and South America in roughly equal proportions (Table 3). Specifically amongst 51 participants from North America with single-strain infection, 31 (61%), 16 (31%) and 4 (8%) had the SD90e, G19080, and HG52 genotypes, respectively. For the 49 participants from South American, the distribution was 31 (63%), 16 (33%), and 2 (4%) respectively for these genotypes, and for the 70 donors from Africa with single-strain infection, the distribution was 45 (57%), 17 (24%), and 8 (11%). Asian participants, limited to 8 from Japan^14^, each had the SD90e genotype. Amongst these 178 participants with the 3 prevalent *UL39* genotypes, there was no association between continent and *UL39* genotype (p = 0.30 or p = 0.58 excluding or including Japanese specimens, respectively).

### Distribution of recombinant strains in persons of known HIV status

Amongst the 183 persons donating specimens with a single *UL39* genotype and with known geographic origin, we examined the distribution of *UL39* genotypes and HIV infection status (Table 3). Amongst these persons, 13 had unknown HIV status, leaving 170 participants, and within these, 5 were infected with HSV-2 strains with rare *UL39* genotypes, leaving 165 persons (83 HIV-uninfected and 82 HIV co-infected) infected with one of the three prevalent *UL39* genotypes. Each major *UL39* genotype were detected in both HIV-uninfected and HIV-infected pesons in roughly equal proportions. Amongst the 83 HIV-uninfected persons, 50 (60%), 25 (30%) and 8 (10%) were infected with the SD90e, G19080, and HG52 genotypes, respectively. Among 82 the HIV co-infected persons, 53 (65%), 23 (28%), and 6 (7%) were infected with SD90e, G19080, and HG52 genotypes, respectively. There was no apparent association between HIV infection status and the three prevelant *UL39* genotypes (p = 0.51). For *UL30* (discussed below), 7 of 72 (9.7%) HIV co-infected and 2 of 66 (3%) HIV-uninfected persons with available data had the variant (p = 0.29). For *UL29* (also discussed below), only one person had the rare variant such that HIV co-infection correlates were not meaningful.

### Co-infection with HSV-2 *UL39* variants

Within-person dual-strain HSV-2 infection is required for HSV-2 × HSV-2 recombination, such as those implied by the data discussed above. A rectal swab from a HIV-infected Peruvian man, R-103-1010 had multiple C-terminal heterozygous *UL39* loci detected by NGS, manual sequencing of bulk *UL39* PCR product, and clonal analysis of amplicons cloned from PCR product (SI Fig. 2, Supplementary Table 6). The pattern indicated a simultaneous, dual HSV-2 SD90e-group and G19080-group infection pattern. *UL39* bulk PCR amplicon sequencing of 10 additional swabs collected over a 14 day genital HSV-2 outbreak were also each heterozygous, indicating a prolonged episode of simultaneous dual-strain infection. Separately, we found that HIV un-infected North America participant 11848 had either a pure SD90e group- or a pure G19080-group *UL39* ddPCR pattern in swab DNA samples collected 4 years apart (SI Table 6), consistent with dual strain infection but not simultaneous reactivation.

### HSV-2 *UL30* and *UL29* genes with HSV-1 × HSV-2 recombination

*UL30* encodes DNA polymerase with mutations conferring drug resistance^21^. It is required for viral replication, has a proof-reading exonuclease function, and cooperatives with *UL42* processitivity factor. Burrel *et al*. sequenced a C-terminal *UL30* variant, termed HSV-2v, from cultured isolates from HIV co-infected West Africans^5^. We sequenced *UL30* in Ugandan specimen 5073_9333, using target capture to enrich HSV DNA (Greninger *et al*. in preparation), and obtained sequence essentially identical to HSV-2v (SI Table 6). We found HSV-2v in 9 (6%) of 146 persons using a ddPCR assay data (SI Table 4). These included 6 of 49 Eastern or Southern African, 1 of 46 North American, and 2 of 51 South American subjects. HIV co-infection was present in 7 persons, but absent in one Ugandan, and in the US subject. The American participants with HSV-2v *UL30* had the G19080 HSV-1 *UL39* insert, while each African had the SD90e group *UL39* gene.

**Figure 4:**
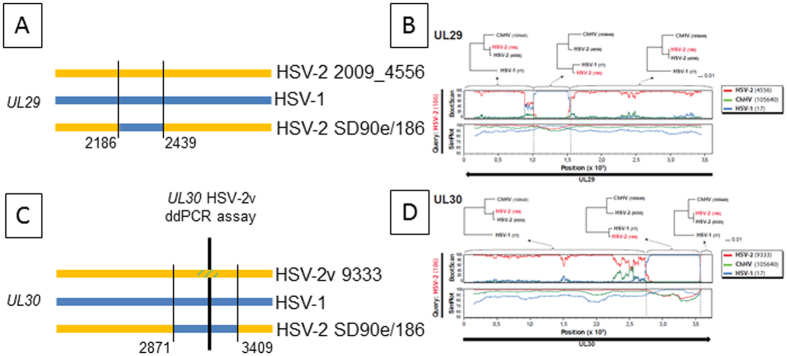
HSV *UL29* and *UL30* genotypes in strains circulating in humans. (**A** and **C**) schematic diagrams of C-terminal coding sequences to approximate scale. HSV-1 is blue, rare HSV-2 strains are yellow. Blue bars in HSV-2 SD90e/186 represent circulating HSV-2 strains that have identity to HSV-1 in this region. Thin vertical black lines and associated strain 186 nucleotide numbers mark lateral flanks of HSV-1 identity. For *UL30*, thick vertical bar and yellow/green hatched zone within HSV-2v 9333 *UL30* represent the locus detected by a ddPCR assay for which strain HSV-2v 9333 contains a variant nucleotide. (**B**,**D**) Recombination analysis of the *UL29* and *UL30* genes in HSV-2 strain 186. Bootscan and Simplot analyses are depicted for each gene. Clear shifts in bootstrap values supporting different phylogenetic topologies indicate recombination crossovers in both genes (also indicated by dotted lines). These crossovers are supported by the Simplot analysis, which demonstrates a shift in similarity with a higher similarity to HSV-1 in the recombination fragments. To further test and visualize ancestry of the recombination fragments, phylogenetic trees based on the recombination fragment and flanking regions are shown. HSV-2 strain 186 clearly shifts from clustering closely to HSV-2 in the trees based on the flanking regions, to clustering closely to HSV-1 in the trees based on the recombination fragments. These results suggest recombination with HSV-2 as major parental, and HSV-1 as minor parental strains.

Burrel *et al*. proposed that HSV-2v *UL30* resulted from HSV-2-ChHV recombination^5^. We aligned full length *UL30* from HSV-1 (n = 98), ChHV^22^, HSV-2v, HSV-2 NGS sequences^13^,^14^,^15^,^16^, laboratory strains, and HSV-2 sequences in Genbank. HSV-1 *UL30* were closely related, with maximum pairwise uncorrected nucleotide distance of 0.01 and little C-terminal variation. HSV-1 17+ and HSV-2 186 were chosen as the species genotypes for recombination analyses for consistency with our analyses of *UL39*. For both *UL30* and *UL29*, strains 186 and the proposed prototype strain SD90e were extremely similar. We noted that most HSV-2 *UL30* sequences have a 539 bp zone of identity to HSV-1 (Table 2, Fig. 4C). This zone contains over 70 consecutive HSV-1-specific allelic SNPs and an HSV-1-specific 12 bp insert. Lateral to this zone, all HSV-2 *UL30* genes, including HSV-2v, are similar, and quite dissimilar from HSV-1 (Fig. 4D). Contravening the ChHV hypothesis, many SNPs and a 3 bp indel in the 539 bp region separate HSV-2v from ChHV. Bootscan (Fig. 4D) confirmed a HSV-1 × HSV-2 crossover event with highly shifting bootstrap values supporting different phylogenetic topologies. Simplot analysis further supported recombination by significant shifts in sequence similarity. Phylogenetic analyses of discrete regions of *UL30* demarcated by the putative crossover zones associated with the tree clustering patterns (Fig. 4D). The phi-statistics (p = 0.0) and RDP4 algorithms demonstrated high significance for recombination (p < 7.6 × 10^−18^ for all methods). Most contemporary HSV-2, exemplified by strains SD90e and 186, have an HSV-1 *UL30* insert. We hypothesize that *UL30* HSV-2v may retain an older sequence related to ChHV.

HSV *UL29* encodes a DNA binding protein, ICP8, involved in DNA unwinding and origin of replication processes, interacting with multiple viral and host proteins. HSV-1 *UL29* sequences (n = 81) were very similar, with a maximum nucleotide distance of 0.0097 and no indels. Alignments of HSV-2 *UL29* disclosed one divergent sequence, 2009_4556, from a 30 year old HIV-infected Kenyan (Fig. 4A). Phylogenetic analyses (Fig. 4B) paralleled *UL30*. Between bp 2186 and bp 2439, HSV-2 186 and SD90e, and other HSV-2 strains show near-identity to HSV-1, including 19 consecutive HSV-1-like alleles. This 254 bp segment placed HSV-1 in the same group as the majority HSV-2 genotype (Fig. 4B). This single unique strain contained 10 differences from ChHV in this region, arguing against origination through recombination with ChHV. Lateral to this region, all HSV-2 were similar but not identical to ChHV and quite divergent from HSV-1. Similar to *UL39* and *UL30*, the bootscan, Simplot, and segmental phylogenetic analyses of *UL29* showed distinct shifts in phylogenetic topologies and sequence similarity, consistent with HSV-1 × HSV-2 recombination. Recombination was statistically supported by the phi-statistics (p = 4.4 × 10^−10^) and the RDP4 program (p < 5.9 × 10^−6^ for all methods except RDP, which did not detect recombination). We propose that an ancestral HSV-1 × HSV-2 *UL29* crossover has become near-fixed in HSV-2, with 2009_4556 as the only currently known strain perhaps retaining an ancestral sequence.

## Discussion

We document HSV-2 strains containing regions of HSV-1 sequence identity within three distinct nucleotide metabolism genes, *UL29*, *UL30*, and *UL39,* involved in HSV virulence, replication, and drug activity. The strains likely arose from interspecies recombination. The principle of HSV-1 × HSV-2 recombination *in vivo* in the natural host has now been established. These findings have implications for viral evolution, HSV drug therapy, vaccines, and viral vector safety.

*UL30* is targeted by HSV drugs^23^ and *UL29* and *UL39* are also antiviral targets^24^,^25^,^26^,^27^,^28^. While *UL30* HSV-2v strains are drug-sensitive^23^, our data suggest that resistance alleles could disseminate via recombination, including across the HSV-1/HSV-2 divide. Attenuated HSV-2 strains^29^ used as vaccines are also of potential concern. These could recombine with HSV-1 to yield replication-competent variants with unexpected properties. Similarly, a replication competent HSV-1 strain expressing GM-CSF is US-licensed for intratumoral injection as oncolytic therapy, at up to 4 × 10^8^ infectious units. Recurrent HSV-2 replication can occur at widespread anatomic areas, including within the central nervous system to cause recurrent meningitis^30^. The possibility of recombinants bearing HSV-2 virulence and replication genes and GM-CSF may be possible has been previously discussed^31^. There is no reason to think that gain of GM-CSF by a wild-type HSV-2 strain would be lead to a selective advantage or increase pathogenicity. Of potentially higher concern, some of the genes deleted in the cancer therapy HSV-1 strain to reduce virulence could be regained, as their HSV-2 versions, via interspecies recombination.

HSV-1 and HSV-2 have separate, overlapping ecologic niches. HSV-1 lytic infection typically involves oral or ocular regions, with latency in trigeminal ganglia. HSV-2 lytic replication is commonest in the anogenital region, with latency in lumbosacral ganglia. Alphaherpesviruses in non-human primates typically show one of these anatomic preferences. The genes responsible for tropism are poorly understood. Remarkably, primary HSV-1 infection now accounts for the majority of cases of clinical first episode genital herpes in the US and northern Europe, while in earlier decades, HSV-2 predominated^32^,^33^. An RFLP search for HSV-1 × HSV-2 IRV amongst HSV-1 strains recovered from human recurrent genital infections did not reveal insertions of HSV-2 sequence^34^. Thus far, analysis of wild-type, low-passage HSV-1 strains by NGS has not revealed insertion of HSV-2 sequences^35^,^36^,^37^, but NGS-based examination of genital HSV-1 strains has not been performed.

HSV-1 × HSV-2 IRV can occur after cell co-infection *in vitro.* Complex patterns of multiple crossovers are typically observed within progency strains. There is some sequence preference for inclusion of origins of replication^3^,^38^ in crossed-over segments. Both these spontaneous IRV, and engineered IRV that are crossed over at desired loci, have been widely used to map species-specific phenotypes (for example^39^,^40^). The large number of such studies cannot be reviewed in detail. Regarding anatomic tropism, *in vivo* research using engineered HSV-1 × HSV-2 IRV suggests segregation of tropism with DNA encoding latency-associated transcripts^41^. Thus far, we have not detected naturally occurring IRV in these loci. Studies *in vivo* suggest that IRV generally have attenuated virulence^42^,^43^. In contrast the IRV described in this report by definition are virulent for infection of humans. IRV can occur *in vivo* after experimental simultaneous HSV-1 and HSV-2 co-inoculation of animals^44^. All animal models have a fundamental differences in pathogenesis compared to HSV infection of humans, for example acute lethality in some systems, and lack of recurrences in almost all. Given the exquisite host-pathogen co-evolution of HSV with humans, demonstration the HSV-1 × HSV-2 IRV occur in the natural host establish a new mechanism for HSV evolution.

We hypothesize that all variants of HSV-2, as well as ChHV, are descended from a common ancestor of HSV-2/ChHV, estimated to have infected a primate around 2 million years ago, rather than by HSV-2/ChHV recombination^5^. A recent estimate places human/chimp divergence at 13 million years ago^45^. We cannot date the HSV-2 × HSV-1 recombination events, but based on the relatively equal geographic distribution of the major *UL39* variants, each appears to predate human emigration from Africa (now estimated at about 70,000 years ago^46^). Recent horizontal transmission of *UL39* variants is also possible. Resolution will require larger, curated specimen sets including those from isolated populations, and optimally archival DNA from before the era of widespread travel. In preliminary analyses of phylogenetic networks, we studied the HSV-1 *UL39* insert found in HSV-2 strain G19080 and the corresponding region from known HSV-1 strains. No clustering into clades was observed as seen for HSV-1 *US7* or *US8*. Interestingly, the HSV-2 strain 19080 *UL39* HSV-1 fragment does not cluster more distantly or closely to any particular HSV-1 strain than do HSV-1 strains cluster with each other. This implies that the HSV-1 *UL39* fragment in HSV-2 G19080 was incorporated into this strain through homologous recombination after emergence of the most recent common ancestor of circulating HSV-1 strains, estimated to have occurred 700,000 years ago^4^.

After their origination, IRV have had different fates in the population. Within *UL29* and *UL30*, small minorities of strains lack the HSV-1-like regions, likely related to fitness. For *UL39*, in contrast, the relatively equal prevalence of the three major genotypes implies that none has a large fitness advantage. It is also possible, especially for *UL29* and *UL30*, that a bottleneck occurred during migration from Africa, as the rare variants are thus far found mostly in Africa.

The grouping of IRV signatures in the 3′ region of HSV *UL39*, including evidence for multiple back-crosses, suggests an ancestral recombination leading to the G19080 group, and susceptibility to back-recombination in this area. It is possible that the recombination points that flank the HSV-1 insert within the HSV-2 G19080 group arose multiple times due to strong site preferences for recombination. Sequence bias for HSV IRV formation has been noted *in vitro*^3^, but the mechanism is unknown. In this earlier work, crossovers were noted near *UL39* but not mapped precisely. The HSV origin of replication in the HSV unique long (UL) region is between *UL29* and *UL30,* but it is not known if this is related to IRV formation. A correlation was found between high GC content and intratypic recombination for HSV-1^47^. HSV-1 *UL29*, *UL30*, and *UL39* GC content very near the average of 66.8% for HSV-1 UL, disfavoring GC content as an explanation. Overall, we consider multiple, independent crossover events less likely than persistence of a relatively ancient recombinant G19080 *UL39* variant in the human population as well as the persistence of the progeny of occasional back-crosses to HSV-2.

Strengths of our approach include the use of direct sequencing, international specimens, the availability of multiple specimens from some subjects to document multi-strain infection, and sequence confirmation with several technologies. HSV sequences can shift *in vitro*^48^ with mutation rates 10–100 fold higher for HSV-2 than HSV-1, and may favor certain genes^49^. *UL39* appears stable *in vitro*: our lab′s strain 333 is identical to that from 3 other labs. The complementary genotyping platforms each have strengths and weaknesses. In-lab progress in HSV-2 target enrichment has recently made direct NGS sequencing practical and economical for swab specimens with scarce HSV-2 DNA and will likely be used in the future.

Our study has several caveats. We may have missed additional IRV within *UL39*, as ddPCR rather than sequencing was used for some specimens. For example, specimen 2008_35742 and specimen 2010_29297 had the same ddPCR patterns (Fig. 2A), but sequencing revealed different 5′ recombination zones. We used a relatively limited set of HSV-2 sequences to detect IRV. Additional full-length HSV-2 sequences including specimens from other continents will likely identify other sites of recombination. Of special interest are detailed studies of specimens from persons with both HSV-1 and HSV-2 infections at the same anatomic site, which might reveal additional and possibly transitory IRV. Our cross-sectional specimen set likely failed to detect some interspecies recombination events that have occurred in the past due to decay of IRV with a fitness disadvantage, and similarly, the temporal sequence of events leading to the variants circulating today cannot be assigned with certainty. Long term, IRV with fitness advantages could reach 100% prevalence, appear as tracts of HSV-1/HSV-2 identity, and again be undetectable without longitudinal samples. The effects of the recombination events detected in this study can be tested *in vitro* or in animals, but it will be difficult to prove their effects on virulence in the natural host.

It has recently been suggested that HSV-2 strain SD90e be used as a prototype, as it is more virulent in animals, is lower passage, and has a functional copy of a gene disabled in HG52^15^. As discussed herein, we elected to use strain 186 as the representative of the most abundant *UL39* genotype for our formal recombination analyses, as strain SD90e contains a rare 9 basepair deletion in *UL39.* Otherwise, the sequences of 186 and SD90e are very similar for the three genes in which we detected interspecies recombination and overall, our data partially support SD90e as an HSV-2 prototype. The selection of a HSV-2 strain as genotypic prototype is complex. Strains 333 and SD90e are identical in the C terminal region of UL39, but the 9 basepair deletion seen in SD90e is not present in 333. We propose that SD90e is a better rational candidate for prototype strain given the fact that 333 is highly passaged and has not been compared head to head with other strains for virulence or protection after administration of candidate vaccines, as has SD90e^18^. For HSV-1, the use of HSV-1 17+ for analyses of *UL29*, *UL30*, and *UL39* is reasonable as there is no consensus as to prototype strain. For the three HSV genes in this report, strain 17+ is closely representative of every HSV-1 sequence data available in Genbank, including those recently analyzed by Szpara *et al*.^35^,^36^,^50^ and of any other HSV-1 strain would have no bearing on the conclusions of this study. This area will likely undergo further refinement as additional sequences and analyses become available.

Functional studies with variant *UL29*, *UL30*, and *UL39* proteins and virus genotypes will be necessary to examine the biological consequences of variation. These genes are pivotal in HSV pathogenesis. Briefly, *UL39* is non-essential *in vitro* but required for virulence *in vivo*, while *UL29* and *UL30* are essential regardless. *UL39* variants could influence dNTP pools and neuronal reactivation, as *UL39* is required for replication in neurons with low endogenous dNTP levels. Additional *UL39* functions include an N-terminal RHIM domain that inhibits necroptosis^51^, a possible protein kinase^52^, and a C-terminal region has been implicated in inhibition of apoptosis. The *UL29* ssDNA binding protein is involved in DNA unwinding and origin of replication processes, interacting with multiple cellular and viral proteins. The *UL30* DNA polymerase has a proofreading exonunclease function and cooperates with the *UL42* processivity factor; mutations can lead to mutator phenotypes and altered drug sensitivity. The variant proteins uncovered herein can be studied biochemically, *in vitro,* or in *vivo* in the isogenic virus context. Within the immunocompetent population there is enormous person-to-person variation in the frequency of HSV-2 symptomatic recurrence and asymptomatic shedding^53^. Quantitative study of reactivation parameters in persons with prevalent IRV genotypes might reveal phenotypic-genotpye correlations.

In summary, contemporary circulating genital HSV-2 strains recovered from widely distributed geographic areas show evidence for recombination with HSV-1. The pattern of *UL39* sequence variants, taken together, is consistent with an ancestral crossover event followed by backcrosses to HSV-2. Data for *UL29* and *UL30* are most consistent with an ancient HSV-1 crossover that has been successfully propagated in the human population, leaving a minority of residual strains with sequences similar to ChHV, the closest known relative of HSV-2. Overall, the principle of HSV-1 × HSV-2 interspecies recombination *in vivo* has now been established in the natural host.

## Methods

### Subjects and specimens

Protocols were approved by the Univerity of Washington Institutional Review Board and all participants provided informed consent. All methods were performed in accordance with the relevant guidelines and regulations for human subjects research. Sources included natural history and interventional protocols HPTN 039 and Partners in Prevention, concerning HSV-2 and HIV transmission^54^,^55^, natural history studies, and a drug treatment study^56^. Subjects and specimen details for NGS have been documented^57^.

### Sequence determination, confirmation, and genotyping

The next-generation sequencing (NGS) laboratory and analysis workflow has been reported^57^ and data uploaded (Genbank accessions KX574861-KX574908 inclusive). BAM Illumina sequence files were assembled and visualized with NCBI Genome Workbench and Integrative Genomics Viewer^58^. Supporting Information Methods contains primers for PCR amplification and dideoxy sequencing of selected regions of HSV-2 DNA (SI Table 5), rapid genotyping by ddPCR (SI Table 4), and Genbank accession numbers (SI Table 6) of new sequences.

### Nucleotide alignment and recombination detection

We accessed full-length ChHV, HSV1 and HSV-2 *UL39* sequences from recent NGS data and other Genbank-available sequences including BLAST searches using HSV-2 HG52 or HSV-1 strain 17 full length *U39* sequences as search terms. Only full-length sequences without ambiguous base calls were used. *UL39* sequences were analyzed full-length or starting at base 1,000 in HSV-2 strain 186 coordinates. Alignments were performed with MegAlign Pro (Lasergene, Madison, WI) using MUSCLE and default parameters. Genetic distances were calculated by the Tamura-Nei method^59^ using Megalign Pro. Phylogenetic trees were generated in MegAlign Pro using default parameters and visualized with Figtree 1.4.2 (http://tree.bio.ed.ac.uk/software/figtree/). Re-rooting for visualization was done within Figtree. Recombination crossovers were identified and visualized using the bootscan method included in the Simplot program. Sequence similarities between different strains were analyzed and visualized using Simplot. The parameter settings for bootscan and Simplot were as follows: Window size: 300 bp, step: 5 bp, gapstrip: on, Kimura (2-parameter), and T/t = 2.0. Detection of recombination, and calculation of statistical significance for recombination, was further performed using the RDP, GENECONV, bootScan, MaxChi, Chimaera, and SiScan algorithms implemented in the RDP4 program^19^ using default settings, and the phi-statistics implemented in the SplitsTree program, after discarding all but one of each set of identical sequences. Phylogenetic trees (networks) based on recombinant fragments, and flanking regions, were constructed using the SplitsTree program and the uncorrected P characters transformation and default settings. Similar analyses were performed for *UL30* and *UL29*.

### Statistics

The abundance of *UL39* genotypes in different continents or among persons with different HIV status was compared with Fisher’s exact test or Chi-squared test, two-tailed.

## Additional Information

**How to cite this article:** Koelle, D. M. *et al*. Worldwide circulation of HSV-2 × HSV-1 recombinant strains. *Sci. Rep.* **7**, 44084; doi: 10.1038/srep44084 (2017).

**Publisher's note:** Springer Nature remains neutral with regard to jurisdictional claims in published maps and institutional affiliations.

## Supplementary Material

Supplementary Information

## Figures and Tables

**Table 1:** Participants contributing specimens for *UL39* genotyping^1^.

Continent	Persons^2^	Sex			HIV-1 infection		
		Men	Women	Unknown	Positive	Negative	Unknown
Prior reports^3^							
North America	9	2	5	2	1	6	2
South America							
Africa^4^	16	4	8	4	5	8	3
Asia	8			8			8
unknown	8			8			8
subtotal	41	6	13	22	6	14	21
Newly reported							
North America	46	22	24		9	37	
South America	50	19	30	1	33	17	
Africa	56	26	30		37	19	
subtotal	152	67	84	1	79	73	
All reported							
North America	55	24	29	2	10	43	2
South America	50	19	30	1	33	17	
Africa	72	30	38	4	42	27	3
Asia	8			8			8
unknown	8			8			8
Total	193	73	97	23	85	87	21

^1^Each individual with *UL39* genotyping information is listed only once regardless of the presence or absence of detection of dual strain infection.

^2^No entry is made for values of zero participants in the indicated demographic groups.

^3^Includes laboratory strain HG52, 333, and 186, strains SD90e and SD66^15^, and strains from Brandt *et al*.^16^.

^4^Includes strains SD90e and SD66.

**Table 2:** Characteristics of circulating HSV-2 genotypes with HSV-1 DNA inserts.

Gene	Strain^1^	5′ HSV-2 marker^2^	5′ HSV-1 marker^3^	3′ HSV-1 marker^3^	3′ HSV-2 marker^2^	Minimum HSV-1 insert length^4^	Sample origin
*UL39*	SD90e	A2370	NA^5^	NA	C2911	NA	N America
*UL39*	G19080	A2370	C2365	G2821	C2911	457	N America
*UL39*	HG52	A2370	C2365	C2536	C2617	172	Europe
*UL39*	2008_35742	A2554	A2548	G2821	C2911	274	N America
*UL39*	2010_29297	C2455	A2465	G2821	C2911	357	N America
*UL29*	2009_4556	G2181	NA	NA	C2451	NA	Kenya
*UL29*	SD90e	G2181	G2196	A2439	C2451	244	N America
*UL30*	5073_9333	C2859	NA	NA	G3444	NA	Kenya
*UL30*	SD90e	C2859	C2856	T3394	G3444	539	N America

^1^Proposed index strain for subgroup. Representative strain if several have the same pattern.

^2^Nucleotide number of HSV-2-specific base in HSV-2 strain 186 numbered from ATG start in relevant gene.

^3^Nucleotide number of HSV-1-specific base in HSV-1 strain 17+ numbered from ATG start in relevant gene.

^4^Minimum number of HSV-1 nucleotides present in the indicated HSV-2 strain.

^5^Not Applicable.

**Table 3:** Geographic distribution and HIV co-infection status of study participants with single defined HSV-2 *UL39* genotypes and known geographic origin.

*UL39* genotype	Continent				Total N (%)
	North America	South America	Africa	Asia	
HSV-2 SD90e/186-like	31	31	45	8	115
HIV co-infected	4	20	29	0	53 (46%)
HIV un-infected	25	11	14	0	50 (44%)
HIV infection status unknown	2	0	2	8	12 (10%)
G19080 long HSV-1 insert	16	16	17	0	49
HIV co-infected	6	11	6	0	23 (49%)
HIV un-infected	10	5	10	0	25 (49%)
HIV infection status unknown	0	0	1	0	1 (2%)
HG52 short HSV-1 insert	4	2	8	0	14
HIV co-infected	0	1	5	0	6 (43%)
HIV un-infected	4	1	3	0	8 (57%)
rare *UL39* genotypes	3	0	2	0	5
HIV co-infected	0	0	2	0	2 (40%)
HIV un-infected	3	0	0	0	3 (60%)
total	54	49	72	8	183
