# Supplementary material for: Worldwide circulation of HSV-2 × HSV-1 recombinant strains
Source: Sci Rep. 2017 Mar 13;7:44084. doi: 10.1038/srep44084 (PMC5347006; doi:10.1038/srep44084)

Supplementary Information

Worldwide circulation of HSV-2 x HSV-1 recombinant strains

David M. Koelle, Peter Norberg, Matthew P. Fitzgibbon, Ronnie M. Russell, Alex L. Greninger, Meei-Li Huang, Larry Stensland, Lichen Jing, Amalia S. Magaret, Kurt Diem, Stacy Selke, Hong Xie, Connie Celum, Jairam R. Lingappa, Keith R. Jerome, Anna Wald, Christine Johnston

Koelle *et al.* Supporting Information

Supporting Information Methods

<u>Subjects and Specimens.</u> Genital swab were obtained and DNA prepared as published (1). Specimens were initially analyzed for the presence of HSV DNA using real-time PCR. The initial PCR detects a type-common target in HSV gene *UL27* that is identical between HSV-1 and HSV-2 (1). The HSV species in positive specimens was then typed using published (2) type-specific real-time PCR targeting HSV type-specific sequences in gene *US4*. Sources of swabs included HPTN039, a study of the effect of acyclovir on HIV-1 acquisition by HSV-2-infected persons (3), Partners in Prevention, a study of the effect of acyclovir on HIV-1 transmissibility by HIV-1/HSV-2 co-infected persons (4), and natural history and drug treatment studies in Seattle and Portland, USA including a study of pritelivir, a candidate anti-HSV drug (5).

<u>Sequencing.</u> HSV-2 lab strains HG52, 333, and 186 are in long-time use in our lab, while HSV-2 lab strain G was newly obtained from ATCC (Manassas, VA). Virus was cultured in Vero cells and isolated as described (6). *UL39* was PCR-amplified with primers UL39wholeprimerF_86940-86959 and UL39wholeprimerR_90508-90526, and dideoxy-sequenced with primers F-HX1 through F-HX5 and R-HX1 (SI Table 6). For specimen 2010_29297 from donor 5060, DNA was similarly amplified and sequenced with primer UL39VarF (SI Table 5). For specimen 2008_35742 from donor 6376, C-terminal *UL39* was amplified with the UL39 Var primer pair (SI Table 5) and sequenced with the same primers. To investigate mixed strain shedding, specimens from Peru332 R-103-1010 were amplified with the UL39Var-primer pair and bulk amplicons either sequenced using the same primers or cloned (TOPO-TA kit, Invitrogen, agCarlsbad, CA) and plasmid DNA from 9 random bacterial colonies

sequenced with primer M13F-21 (SI Table 5). NGS used the Illumina platform. The protocol for direct sequencing of swab DNA without target enrichment has been described separately (7). For selected swab DNA specimens, HSV-2 DNA was enriched using a custom biotinylated 120bp oligonucleotide panel (IDT, Coralville, IA) tiling across HSV-2 strain HG52 Genbank NC_001798 genome (Greninger *et al.* in preparation). For these samples, libraries were created using Fragmentase™ (New England Biolabs, Ipswich, MA), *E. coli* DNA polymerase, T4 polynucleotide kinase, and Taq DNA polymerase (8), followed by amplification with barcoded TruSeq™ (Illumina, San Diego, CA) primers and pooling prior to enrichment. Libraries were enriched using the manufacturer's reagents and protocols (IDT) and sequenced using a single ended v3 150bp kit and MiSeq instrument (Illumina). Non-enriched sequence reads were filtered by discarding human reads and aligning to the HSV-2 HG52 genome while post-enrichment libraries were aligned directly the HG52 genome.

HSV genotyping by digital droplet PCR (ddPCR). Two reactions were used for *UL39* and one for *UL30*. The first *UL39* reaction resolved HSV type in the 5' portion of the long *UL39* recombination (see main text). Primers and probes (SI Table 4) allowed differentiation of HSV type in the vicinity of HSV-2 *UL39* bp 2415-2428 (coordinates from Genbank M12700.1 for strain HSV-2 186) from the homologous but different HSV-1 sequence in the vicinity of *UL39* bp 2394-2407 (Genbank JN555585.1 for strain HSV-1 17+). The second *UL39* reaction resolved HSV type towards the 3' end of the long *UL39* recombination. Primers and probes specified type-specific signals in the vicinity of HSV-2 *UL39* bp 2671-2683, homologous to but different from HSV-1 sequence in the vicinity of *UL39* bp 2650-2662 region. The *UL30* reaction differentiated between bp 3000-3100 of the HSV-2 strain 186 sequence from almost identical regions of HSV-2v. For each reaction, 25 μl mixes were assembled from 12.5 μl ddPCR SI 2xSupermix (BioRad), 500 nM each primer, 250 nM each probe and 10 μl DNA. Twenty μl was

used to generate droplets that were amplified, analyzed and counted. The amplification thermal cycling profile was 94 ^{o}C for 10 minutes, followed by 45 cycles of 94 ^{o}C for 30 seconds and 60 ^{o}C for 1 minute, then hold at 98 ^{o}C for 10 minutes followed by 4 ^{o}C until analyzed. For each reaction, VIC and FAM fluorescence indicated distinct HSV genotypes (see main text).

References for Koelle *et al.* Supporting Information

1. Jerome KR, Huang ML, Wald A, Selke S, & Corey L (2002) Quantitative stability of DNA after extended storage of clinical specimens as determined by real-time PCR. *Journal of clinical microbiology* 40(7):2609-2611.
2. Corey L, Huang ML, Selke S, & Wald A (2005) Differentiation of herpes simplex virus types 1 and 2 in clinical samples by a real-time taqman PCR assay. *Journal of medical virology* 76(3):350-355.
3. Celum C*, et al.* (2008) Effect of aciclovir on HIV-1 acquisition in herpes simplex virus 2 seropositive women and men who have sex with men: a randomised, double-blind, placebo-controlled trial. *Lancet* 371(9630):2109-2119.
4. Celum C*, et al.* (2010) Acyclovir and transmission of HIV-1 from persons infected with HIV-1 and HSV-2. *The New England journal of medicine* 362(5):427-439.
5. Wald A*, et al.* (2014) Helicase-primase inhibitor pritelivir for HSV-2 infection. *The New England journal of medicine* 370(3):201-210.
6. Koelle DM*, et al.* (2001) CD8 CTL from genital herpes simplex lesions: recognition of viral tegument and immediate early proteins and lysis of infected cutaneous cells. *Journal of immunology* 166(6):4049-4058.
7. Johnston C, Magaret, A., Diem, K., Fitzgibbon, M, Huang, M., Selke, S., Rashevsky A., Lingappa, J.R., Celum, C., Koelle, D.M., Wald, A. (Sequence heterogeneity in herpes simplex virus type 2 identied using next generation sequencing. *In preparation*.
8. Salipante SJ*, et al.* (2015) Characterization of a multidrug-resistant, novel Bacteroides genomospecies. *Emerging infectious diseases* 21(1):95-98.

Supporting Information Table 1. Alternative names, Genbank and literature references of previously reported HSV-2 *UL39* sequences analyzed.

Reference, name in this report	Previous name(s)	Continent	Genbank accession	Pubmed PMID number of literature citation for sequence
Lab strains				
186	None	N America	JX112656.1	5694774, none for sequence
SD90e	None	Africa	KF781518.1	24503076
HG52	None	Europe	JN561323.2	None, update of 9499055
333	None	N America	KP192856.1	25855744
Kolb *et al.* 2015				
1192	None	N America	KP334095.1	25855744
GSC-56	None	N America	KP334094.1	25855744
CtSF	None	N America	KP334097.1	25855744
CtSF-R	None	N America	KP334093.1	25855744
COH 3818	None	N America	KP334096.1	25855744
Newman *et al.* 2015				
G19070	8937_1999_3336	N America	KR135298	26018166
G19083	10883_2001_13347	N America	KR135311	26018166
G19084	9335_2005_576	N America	KR135312	26018166
G19085	9335_2007_14	N America	KR135313	26018166
G19086	7444_1996_25809	N America	KR135314	26018166
89_350_MA__KR135321	89_390	N America	KR135321	26018166
G19080	44_619833	N America	KR135308	26018166
G19081	44_419851	N America	KR135309	26018166

G19082	44_319857	N America	KR135310	26018166
BethesdaP5 KR135330	BethesdaP5	N America	KR135298	26018166
G19071	M22987	Africa	KR135299	26018166
G19072	D30613	Africa	KR135300	26018166
G19073	F70764	Africa	KR135301	26018166
G19074	M1119	Africa	KR135302	26018166
G19075	L22861	Africa	KR135303	26018166
G19076	H00066	Africa	KR135304	26018166
G19077	K39924	Africa	KR135305	26018166
G19078	A76191	Africa	KR135306	26018166
G19079	J09622	Africa	KR135307	26018166
G19087	J32715	Africa	KR135315	26018166
G19088	G75809	Africa	KR135316	26018166
G19089	A76832	Africa	KR135317	26018166
G19090	D39650	Africa	KR135318	26018166
G19091	D39765	Africa	KR135319	26018166
SD66 KR135320	SD66	Africa	KR135320	26018166
G32586_UL39_JA1	JA1	Asia	KR135322	26018166
G32587_UL39_JA2	JA2	Asia	KR135323	26018166
G32588_UL39_JA3	JA3	Asia	KR135324	26018166
G32589_UL39_JA5	JA5	Asia	KR135325	26018166
G32590_UL39_JA6	JA6	Asia	KR135326	26018166
G32591_UL39_JA7	JA7	Asia	KR135327	26018166
G32592_UL39_JA8	JA8	Asia	KR135328	26018166

G32593_UL39_JA9	JA9	Asia	KR135329	26018166
Various	Not applicable	Various	KX574860 through KX574908, inclusive	Not applicable

Supporting Information Table 2. HSV-2 *UL39* length variation and amino acids indels relative to strain 186.

representative strain	location of indel relative to HSV-2 strain 186 *UL39*	change	length, AA	Genbank
186	not applicable	not applicable	1144	JX112656
SD90e	between S118 and A122	del GPS[1]	1141	KF781518
G	between S118 and A122	del GPS[1]	1141	KT992822
HG52	between G205 and S208	del SG[2]	1142	JN561323
D39650	between G275 and T276	ins AG[3]	1146	KR135318

[1]Deletion is ambiguous as strain 186 sequence is SGPS in this region. Change could also be recorded as deletion SGP. In strain SD90e, one S is retained.

[2] Deletion is ambiguous as strain 186 sequence is GSG in this region. Deletion could also be recorded as deletion GS. In strain HG52, one G is retained.

[3] Insertion is ambiguous as strain 186 sequence is GAG in this region. In strain D39650, GAG peptide expands to GAGAG.

Supporting Information Table 3. SNPs within the HSV-1 inserts in HSV-2 *UL39* sequences.

	Prototype virus species and strain		Strains representative of prevalent *UL39* IRV clades with single HSV-1 inserts				Rare strains with unique *UL39* genotypes with single HSV-1 insets				Rare strains with unique *UL39* genotypes with complex, multiple HSV-1 inserts or crossovers					
			G19080[4] long insert		HG52 short insert		2008_35742[4] short insert		2010_29297[4] short insert		G19083[4]		F_2009-4556[4]		F_2009-2198[4]	
Location in *UL39* consensus[1]	HSV-2 186[3]	HSV-1 17[3]	Allele	Genotype in prototype strain	Allele	Genotype in prototype strain	Allele	Genotype in prototype strain	Allele	Genotype in prototype strain	Allele	Genotype in prototype strain	Allele	Genotype in prototype strain	Allele	Genotype in prototype strain
5' loci[2]				2				2		2		2		2		2
2417	A	g	A	2	A	2	A	2	A	2	A	2	A	2	A	2
2429	T	c	c	1	c	1	T	2	T	2	c	1	c	1	T	2
2450	G	c	c	1	c	1	G	2	G	2	G	2	c	1	G	2
2466	T	g	g	1	g	1	T	2	T	2	T	2	g	1	T	2
2470	G	c	c	1	c	1	G	2	G	2	c	1	c	1	c	1
2474	A	g	g	1	g	1	A	2	A	2	A	2	g	1	A	2
2477	C	a	a	1	a	1	C	2	C	2	C	2	a	1	C	2
2483	C	t	t	1	t	1	C	2	C	2	C	2	t	1	C	2
2501	C	a	a	1	a	1	C	2	C	2	a	1	a	1	C	2
2529	C	a	a	1	a	1	C	2	a	1	C	2	C	2	C	2
2533	G	a	a	1	a	1	G	2	a	1	G	2	G	2	G	2
2540	C	t	t	1	t	1	C	2	t	1	t	1	C	2	C	2
2543	T	c	c	1	c	1	T	2	c	1	c	1	T	2	T	2
2549	C	g	g	1	g	1	C	2	g	1	g	1	C	2	C	2
2550	A	c	c	1	c	1	A	2	c	1	c	1	A	2	A	2
2551	T	g	g	1	g	1	T	2	g	1	g	1	T	2	T	2
2582	A	g	g	1	g	1	A	2	g	1	A	2	A	2	g	1
2588	T	g	g	1	g	1	T	2	g	1	T	2	T	2	T	2
2591	T	c	c	1	c	1	T	2	c	1	T	2	T	2	T	2
2600	A	c	c	1	c	1	A	2	c	1	A	2	A	2	A	2
2612	G	a	a	1	a	1	a	1	a	1	G	2	G	2	a	1
2615	G	a	a	1	a	1	a	1	a	1	G	2	G	2	a	1
2618	G	c	c	1	c	1	c	1	c	1	G	2	G	2	c	1
2631	G	a	a	1	a	1	a	1	a	1	G	2	G	2	a	1
2640	C	a	a	1	a	1	a	1	a	1	C	2	C	2	a	1
2663	C	a	a	1	C	2	a	1	a	1	C	2	C	2	C	2
2666	T	c	c	1	T	2	c	1	c	1	T	2	T	2	T	2
2690	G	c	c	1	G	2	c	1	c	1	G	2	G	2	G	2
2700	A	c	c	1	A	2	c	1	c	1	A	2	A	2	A	2
2705	C	g	g	1	C	2	g	1	g	1	C	2	C	2	C	2
2720	G	t	t	1	G	2	t	1	t	1	t	1	t	1	G	2
2726	G	a	a	1	G	2	a	1	a	1	G	2	a	1	G	2
2729	C	t	t	1	C	2	t	1	t	1	C	2	t	1	C	2
2731	G	a	a	1	G	2	a	1	a	1	G	2	a	1	G	2
2743	C	a	a	1	C	2	a	1	a	1	C	2	a	1	C	2
2768	C	g	g	1	C	2	g	1	g	1	C	2	g	1	C	2
2769	G	t	t	1	G	2	t	1	t	1	G	2	t	1	G	2
2774	C	g	g	1	C	2	g	1	g	1	C	2	g	1	C	2
2786	T	c	c	1	T	2	c	1	c	1	T	2	c	1	T	2
2810	G	c	c	78G	G	2	c	1	c	1	G	2	c	1	G	2
2821	G	a	a	1	G	2	a	1	a	1	G	2	G	2	G	2
2843	C	t	t	1	C	2	t	1	t	1	C	2	C	2	C	2
2846	G	c	c	1	G	2	c	1	c	1	G	2	G	2	G	2
2874	T	c	c	1	T	2	c	1	c	1	T	2	c	1	T	2
2877	A	g	g	1	A	2	g	1	g	1	A	2	g	1	A	2
2883	A	c	c	1	A	2	c	1	c	1	A	2	c	1	A	2

2885	C	g	g 1	C 2	g 1	g 1	C 2	g 1	C 2
2957	C	t	C 2	C 2	C 2	C 2	C 2	C 2	C 2
3' loci[2]			2		2	2	2	2	2

[1] Loci numbers in consensus sequence from aligning the indicated *UL39* sequences with Clustal Omega (Megalign, Lasergene, Inc).

[2] Except for scattered strain-specific SNPs and indels, all loci in the 5' and 3' regions outside of the region detailed in this table, for all HSV-2 strains, have HSV-2 strain SD90e/186-like sequences.

[3] HSV-2 alleles are capitalized and HSV-1 alleles are lower case.

[4] The HIV infection status of the subjects from whom these samples were obtained is G19080: HIV-infected; 2008_35742: HIV-uninfected; 2010_29297: HIV-uninfected; 19083: HIV-uninfected; 2009-4556: HIV-infected; 2009-2198: HIV-infected

Supporting Information Table 4. Primers and probes for digital droplet PCR.

Assay	Amplicon region[1]	Primer 1[2]	Primer 2[2]	Probe(s)[3]	Note
UL39 left	*HSV-2 UL39* bp 2386-**2475**;<br>HSV-1 *UL39* bp 2371-2454	UL39_1_2371_2390<br>AACCTCTGCACSGAGATCGT	UL39_1_2437_2454<br>GCATCGGGCCAGATTCAC	UL39_S_MGBP_1P<br>VIC-TCCGGCCT***C***CAAGC-MGB<br>UL39_S_MGBP_2P<br>FAM-CCCGTCCT***G***CAAAC-MGB[4]	Two type-common primers combined with two differentially labeled type-specific probes in one reaction.
UL39 right	HSV-1 *UL39* bp 2617-2689	UL39_1_2617_2633<br>ACGGCCTGCCTGAAGCT	UL39_1_2671_2689<br>CCTCGGCGATGTGTTTGTT	UL39_1L_MGB_1P<br>VIC-TCTGCCGAATTTC-MGB	Combined with HSV-2 primers and probes in the line below in single reaction with four primers and two probes.
UL39 right	HSV-2 *UL39* bp 2648-2710	UL39_2_2648-2667<br>TGAAGATGGGCCTGGATCTG	UL39_2_2692-2710<br>CCTCGGCGATGTGTGTGTT	UL39_1L_MGB_2P<br>FAM-TCGGCCGAGTTCC-MGB	Combined with HSV-1 primers and probes in the line above in single reaction with four primers and two probes.
UL30	HSV-2 strain 186 *UL30* bp 3004-3116;HSV-1 strain 17 UL30 2989-3101;ChHV *UL30* bp 3016-3128	UL30_HSV2_3004_3018 =<br>UL30_HSV1_2989_3013 =<br>UL30_ChHV_3016_3030<br>GAGTGGCTGGCGCGR	UL30_HSV2_3094-3116 =<br>UL30_HSV1_3079_3101=<br>UL30_ChHV_3106_3128<br>GTGAGGACAAAGTCCTGGATGTC	HSV_1_UL30_PB30496<br>FAM-ACTGCAGGCGTTCG-MGB<br><br>HSV_2_CHHV_UL30_PB3049<br>VIC-ACTGCGGGAGTTTGA-MGB	Two virus-common primers combined with two differentially labeled probes in one reaction. The upper probe detects both HSV-1 and majority HSV-2; the lower probe is specific for ChHV and the rare HSV-2 variant (see text)

[1] *UL39* coordinates from HSV-2 strain 186 (Genbank JN561323.2), or HSV-1 strain 17+ (Genbank JN555585.1). *UL30* coordinates from HSV-2 strain HG52 or chimpanzee alphaherpesvirus (Genbank JQ360576.1).

[2] Name of primer followed by 5’ $\rightarrow$ 3’ sequence. Note that underlined S indicates an equimolar mixture of G and C; underlined R is equimolar mix of A and G.

[3] Name of probe followed by 5’ $\rightarrow$ 3’ sequence. VIC = proprietary dye, MGB = minor groove binder quencher dihydrocyclopyrroloindole tripeptide, FAM = 6-carboxyfluorescein. Underlined nucleotides are discriminatory between HSV-1 and HSV-2 or between HSV-1 and ChHV.

[4]There is variation at UL39 nucleotide 2423 (HSV-2 HG52 coordinates) in ***bold italics*** between probe UL39_S_MGBP_1P and probe UL39_S_MGBP_2P. HSV-2 strains 333 and 186, differ at 2423 with 186 having a G nucleotide and 333 having a C nucleotide. HSV-1 strains also have a C at this position. For detection of HSV-2, the presence of the single mismatch at 2423 was tolerated by probe UL39_S_MGBP_2P for identification of ddPCR amplicons from both HSV-2 strains 333 and 186.

Supporting Information Table 5. Primers used for PCR and/or fluorescent dideoxy sequencing.

primer name	sequence 5'→3'	target sequence and comments
UL39 wholeprimerF_8640-86959[1]	CCGGTGCGTCCTTTCGGTCG	full length *UL39* PCR and sequencing
UL39 wholeprimerR_90508-90526	CGGGATCCATGGCGATATG	full length *UL39* PCR and sequencing
F-HX1 87022- 87043	CACCTTGGTTCCAATGGCCA	primer for full length *UL39* sequencing
F-HX2 87757- 87776	ACGTTGTCGTTCGTCGCAGA	primer for full length *UL39* sequencing
F-HX3 88435—88454	GAAAGGCTTCGCGAACACGA	primer for full length *UL39* sequencing
F-HX4 89130- 89149	TCTGGATGCCGGACCTGTTC	primer for full length *UL39* sequencing
F-HX5 89816-89835	CACTTTAAGCGCAGCATGTA	primer for full length *UL39* sequencing
R-HX1 90493- 90474	GATCGGAGCGCTGTTGCTTA	primer for full length *UL39* sequencing
UL39VarF	GGGACACCAGCATGTCGCTCG	PCR and bulk amplicon sequencing for C-terminal region of *UL39* mixed strain specimen
UL39VarR	ACTCCAGATCCAGGCCCATCTT	PCR and bulk amplicon sequencing for C-terminal region of *UL39* mixed strain specimen
M13F-21	TGTAAAACGACGGCCAGT	sequencing primer for plasmid colonies for TA-cloned C-terminal region of *UL39*

[1]Coordinates are from HSV-2 strain HG52 Genbank JN56323.2

Supporting Information Table 6. HSV-2 sequences deposited in Genbank for this report.

subject	strain	demographics	target	Genbank	notes	technology
HSV-2 *UL39* genes with the different genotypes from two swab specimens from the same subject						
VRC11848	2003_16029	HIV+ male age 46 USA	*UL39* full length	KT966384	Same subject as 2007_22031 but different *UL39* allele. Has long, G19080-like HSV-1 *UL39* insert	Manual dideoxy
VRC11848	2007_22031	HIV+ male age 49 USA	*UL39* full length	KT984817	Same subject as 2003_16029 but different UL39 allele Has HSV-2 186-like *UL39*	Manual dideoxy
HSV-2 *UL39* genes with the same genotype from two swab specimens from the same subject						
VRC6921	2006_49895	HIV- male age 24 USA	*UL39* full length	KT966386	Same subject as 2006_49895 and same 186-like *UL39* allele	Manual dideoxy
VRC6921	2006_50074	HIV- male age 24 USA	*UL39* full length	KT966387	Same subject as 2006_50074 and same 186-like *UL39* allele	Manual dideoxy
HSV-2 *UL39* gene with long HSV-1 insert representative of the HSV-2 G19080 group recovered from a swab specimen						
VRC12162	2004_20601	HIV+ male age 31 USA	*UL39* full length	KT966385	Near full length representative of long HSV-1 *UL39* insert G19080 group	Manual dideoxy
HSV-2 *UL39* gene similar to HSV-2 SD90e and 186 recovered from a swab specimen						
VRC6192	2010_21	HIV- female age 71 USA	*UL39* full length	KT984818	Strain 186-like UL39	Manual dideoxy
HSV-2 *UL39* gene segments with rare genotypes recovered from swab specimens						
VRC6376	2008_34536	HIV- male 39 USA	*UL39* C terminal region	KT966390	Has rare short right variant HSV-1 insert	Manual dideoxy
5060	2010_29299	HIV- female 28 USA	*UL39* C terminal region	KU053644	Has rare short right variant HSV-1 insert	Manual dideoxy
1216599501201	2009_2198	HIV+ male age 44 Zambia	*UL39* full length	KU133323	Has rare short right variant HSV-1 insert. Abstracted from SRP057035 read archive	Illumina
1684599501201	2009_4556	HIV+ male age 30 Kenya	*UL39* full length	KU133324	Has rare short right variant HSV-1 insert. Abstracted from SRP057035 read archive	Illumina
Cloned HSV-2 UL*39* amplicons from a single swab with heterozygosity at multiple *UL39* loci						
332 R-103-1010	2004_4373	HIV+ male age 31 rectal Peru	*UL39* C terminal region	KT966388	commoner variant, G19080-like	Manual dideoxy
332 R-103-1010	2004_4373	HIV+ male age 31 rectal Peru	*UL39* C terminal region	KT966389	rare variant, strain 186-like	Manual dideoxy
HSV-2 *UL39* genes from laboratory strains						
Not applicable	HG52	Not applicable lab strain multiply passaged	*UL39* full length	KT984819	Identical to existing Genbank sequence for HG52, has short HSV-1 *UL39* insert. Virus in long-term use in our lab.	Manual dideoxy
Not applicable	333	Not applicable	*UL39* full	KT984820	Identical to existing	Manual dideoxy

		lab strain multiply passaged	length		Genbank sequence for 333. Virus in long-term use in our lab.	
Not applicable	186	Not applicable lab strain multiply passaged	*UL39* full length	KT984821	Identical to existing Genbank sequence for 186. Virus in long-term use in our lab.	Manual dideoxy
Not applicable	G	Not applicable lab strain passaged once	*UL39* full length	KT992822	Virus obtained from ATCC.	Illumina
HSV-2 *UL30* genes or segments						
5073	9333	HIV+ male age 55 Uganda	*UL30* full length	KU258496	Similar to HSV-2v from Burrel *et al.2015* PMID 26401046. Obtained with target enrichment workflow.	Illumina
1684599501201	2009_4556	HIV+ male age 30 Kenya	*UL30* full length	KU886303	Similar to majority HSV-2 genotype. Abstracted from SRP057035 read archive	Illumina
Not applicable	G	Not applicable lab strain passaged once	*UL30* full length	KU133320	Virus obtained from ATCC	Illumina
HSV-2 *UL29* genes or segments						
1684599501201	2009-4556	HIV+ male age 30 Kenya	*UL29* full length	KU133322	Rare variant. Abstracted from SRP057035 read archive	Illumina
Not applicable	G	Not applicable lab strain passaged once	*UL29* full length	KU133321	Virus obtained from ATCC	Illumina
Other sequences relevant to rare HSV-2 strains bearing the HSV-2v *UL30* sequence as identified by Burrel et al. J. Virology 2015 PMID 27401046						
5073	9333	HIV+ male age 55 Uganda	Unique Long (UL) region	KU893109	Preliminary assembly from target enrichment workflow	Illumina

Supplemental Information Figure Legends

SI Fig. 1. A: ddPCR genotyping of HSV-2 swab DNA with 5' (top row) and 3' (lower row) *UL39* locus-specific assays. Colored bars schematize the 3' region of *UL39* with HSV-2 strain 186 in red and HSV-1 inserts in blue. Blue and green droplets with HSV-2 strain 186 and HSV-1 genotype PCR products have Y and X axis fluorescence, respectively. For specimen 2010_29297 from subject 5060, dark black circles contain small numbers of positive droplets. PCR product-negative droplets are black. Cutoffs (purple lines) for positive signals are adjusted for each assay. B-D: Simultaneous infection with HSV-2 strains with different *UL39* genotypes for subject R-103-1010. B: Chromatogram for bulk PCR product is shown below chromatograms for representative cloned HSV-2 186-like and HG52-like variant PCR product. C: ddPCR dotplots for this specimen showing droplets heterozygous at *UL39* loci in upper right quadrants. D: Portion of aligned Illumina NGS for region in B. Vertical arrows connect equivalent genomic positions in sections B and D showing heterozygosity.

SI Fig. 2. Phylogenetic tree of 85 full length HSV-2 *UL39* sequences, HSV-1 strain 17+ *UL39*, and ChHV *UL39.* Selected strains are indicated in larger font.

SI Fig. 3. Recombination analysis of 3' region of the *UL39* gene in HSV-2 strains 2010_29297 (participant 5060) and 2008_35742 (participant 6376). Analysis of HSV-2 strain HG52 is shown at bottom for comparison of the location of 5' recombination crossovers. X axis indicates position in the *UL39* ORF in kilobases starting at ATG start. Bootscan and Simplot analyses are depicted for each strain. Clear shifts in bootstrap values supporting different phylogenetic topologies indicate 5' recombination crossovers in all strains (also indicated by dotted lines), but the locations of the crossovers differ between the strains. Each crossover is supported by the Simplot analysis, which demonstrates a shift in similarity with a higher similarity to HSV-1 in the

recombination fragments. To further test and visualize ancestry of the recombination fragments, phylogenetic trees based on the recombination fragment and flanking regions are shown for these strains. The analyzed strains clearly shift from clustering closely to HSV-2 in the trees based on the flanking regions, to clustering closely to HSV-1 in the trees based on the recombination fragments. 3’ crossovers are not well seen in these analyses due to lack of sequence distal to the *UL39* stop codon. These results suggest recombination with HSV-2 as major parental, and HSV-1 as minor parental strains.

Koelle *et al.* SI Fig. 1

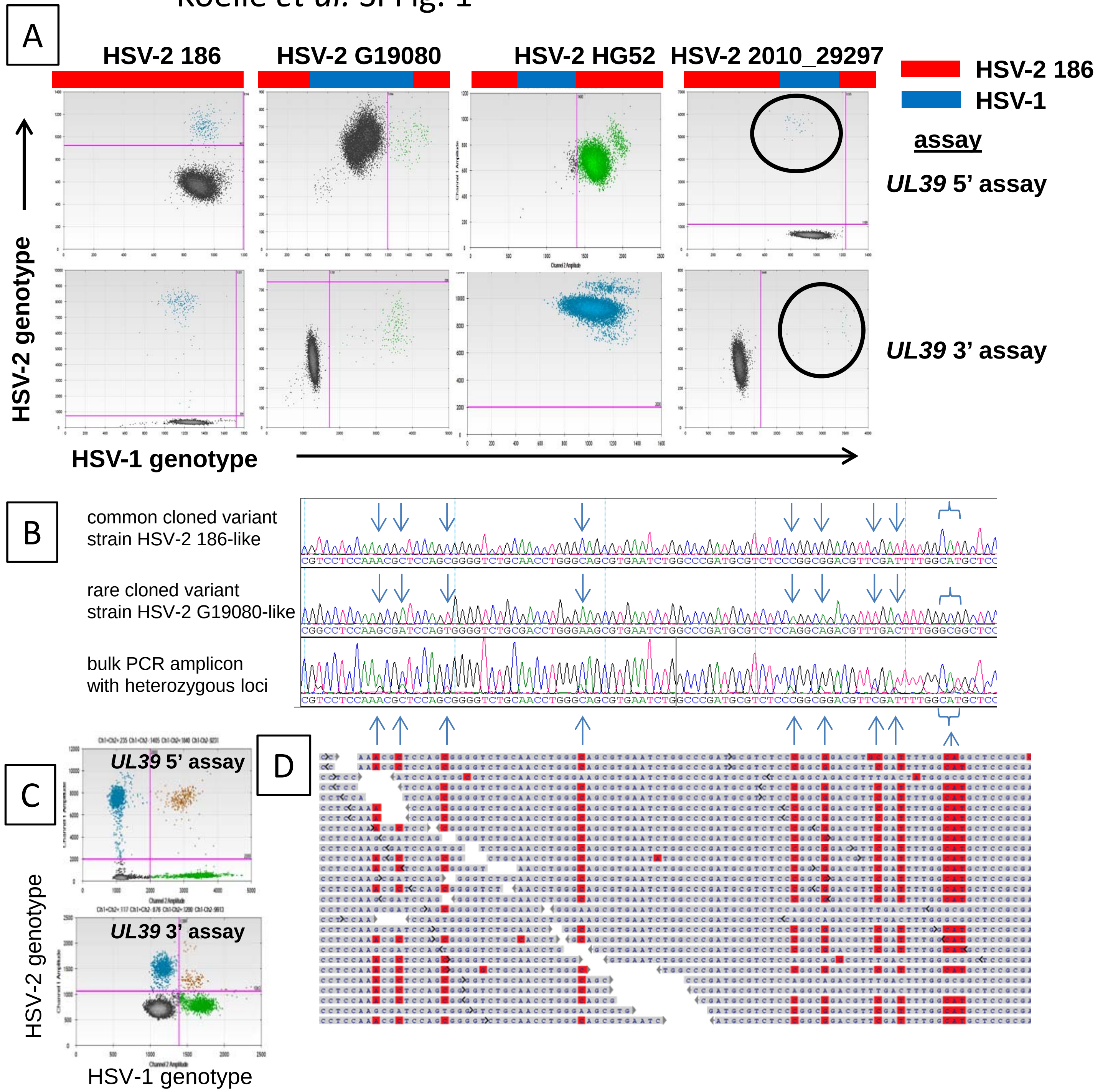

Koelle *et al.* SI Fig. 2

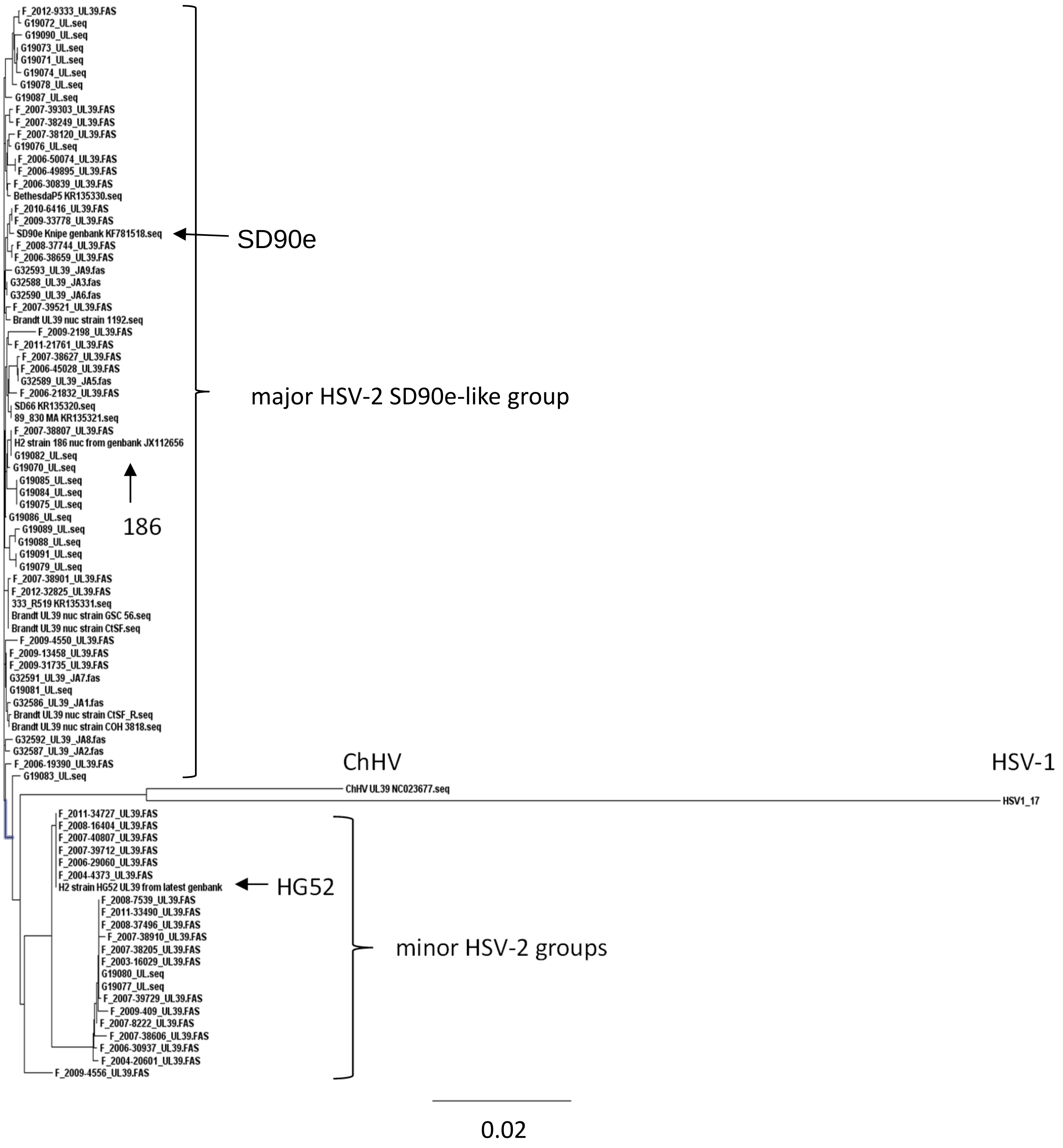

Koelle *et al.* SI Fig. 3

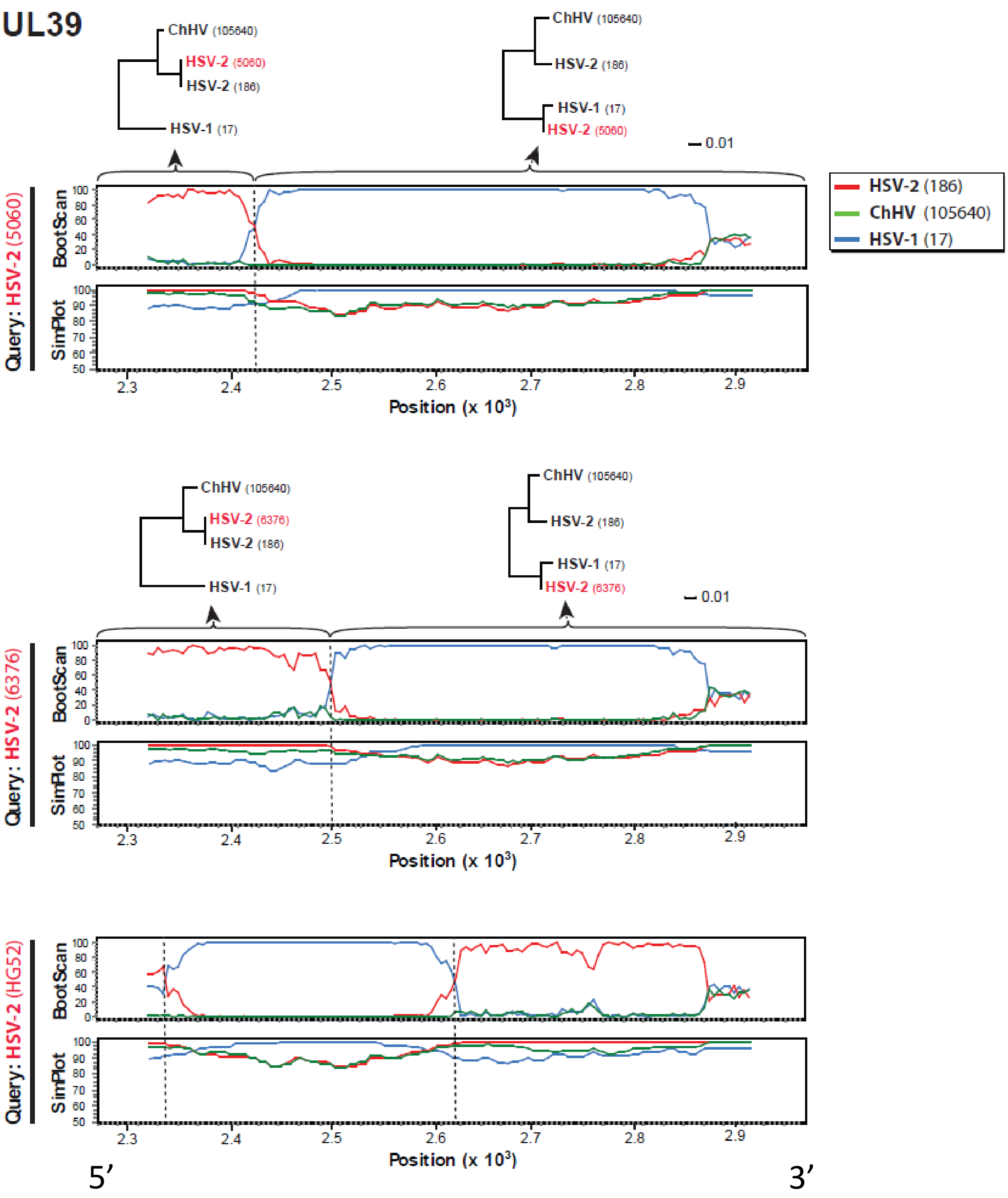

Supplement: Supplementary Information [file srep44084-s1.pdf]
